# Trait Emotional Intelligence in Surgeons

**DOI:** 10.3389/fpsyg.2022.829084

**Published:** 2022-03-10

**Authors:** K. V. Petrides, Matheus F. Perazzo, Pablo A. Pérez-Díaz, Steve Jeffrey, Helen C. Richardson, Nick Sevdalis, Noweed Ahmad

**Affiliations:** ^1^London Psychometric Laboratory, University College London, London, United Kingdom; ^2^School of Dentistry, Federal University of Goiás, Goiânia, Brazil; ^3^Institute of Psychology, Austral University of Chile, Puerto Montt, Chile; ^4^Steve Jeffrey International FZE LLC, Dubai, United Arab Emirates; ^5^Department of Otolaryngology, James Cook University Hospital, Cleveland, United Kingdom; ^6^Centre for Implementation Science, King’s College London, London, United Kingdom

**Keywords:** clinical competencies, occupational profiling, personality, surgical training, military, TEIQue

## Abstract

Trait emotional intelligence (trait EI or trait emotional self-efficacy) concerns people’s perceptions of their emotional functioning. Two studies investigated this construct in surgeons and comparison occupations. We hypothesized that trait EI profiles would differ both within surgical specialties as well as between them and other professions. Study 1 (*N* = 122) compared the trait EI profiles of four different surgical specialties (General, Orthopedic, Head and Neck, and Miscellaneous surgical specialties). There were no significant differences amongst these specialties or between consultant surgeons and trainees in these specialties. Accordingly, the surgical data were combined into a single target sample (*N* = 462) that was compared against samples of engineers, executives and senior managers, lawyers, junior military managers, nurses, and salespeople. Surgeons scored significantly higher on global trait EI than junior military managers, but lower than executives and senior managers, salespeople, and nurses. There were no significant differences vis-à-vis engineers or lawyers. A MANOVA confirmed a similar pattern of differences in the four trait EI factors (Wellbeing, Self-control, Sociability, and Emotionality). Global trait EI scores correlated strongly with single-question measures of job satisfaction (*r* = 0.47) and job performance (*r* = 0.46) in the surgical sample. These findings suggest that interventions to optimize the trait EI profiles of surgeons can be helpful in relation to job satisfaction, job performance, and overall psychological wellbeing.

## Introduction

Trait emotional intelligence (trait EI) is defined as a constellation of emotional perceptions assessed through questionnaires and rating scales (Petrides et al., 2007). Essentially, trait EI concerns people’s perceptions of their emotional abilities, which is why it has also been labeled as “trait emotional self-efficacy.” Table 1 presents the sampling domain of trait EI in adults.

**TABLE 1:** The sampling domain of trait EI in adults.

Factors and Facets	High scorers perceive themselves as…
**Wellbeing**	
Self-esteem	…successful and self-confident.
Trait happiness	…cheerful and satisfied with their lives.
Trait optimism	…confident and likely to “look on the bright side” of life.
**Self-control**	
Emotion regulation	…capable of controlling their emotions.
Stress management	…capable of withstanding pressure and regulating stress.
Impulse control	…reflective and less likely to give into their urges.
**Emotionality**	
Emotion perception	…clear about their own and other people’s feelings.
Emotion expression	…capable of communicating their feelings to others.
Relationships	…capable of having fulfilling personal relationships.
Empathy	…capable of taking someone else’s perspective.
**Sociability**	
Social awareness	…accomplished networkers with excellent social skills.
Emotion management	…capable of influencing other people’s feelings.
Assertiveness	…forthright, frank, and willing to stand up for their rights.
Adaptability*	…flexible and willing to adapt to new conditions.
Self-motivation*	…driven and unlikely to give up in the face of adversity.
Global trait EI	

*These facets are not keyed to any factor, but feed directly into the global trait EI score.

There is an expanding body of evidence (many hundreds of studies), including behavioral genetic investigations (van der Linden et al., 2017, 2018; Alegre et al., 2019), giving reasons to conceptualize trait Emotional intelligence as part of human personality. In addition, numerous studies have revealed theoretically meaningful associations with important outcomes like academic performance at university (Sanchez-Ruiz and El Khoury, 2019), career decision-making (Farnia et al., 2018), positive affect (Di Fabio and Kenny, 2019), body image (Pollatos et al., 2020), and mental health (Petrides et al., 2017; Rudenstine and Espinosa, 2018), among many others. Trait EI shows incremental validity over the Giant Three (Psychoticism, Extraversion, Neuroticism), the Big Five (Neuroticism, Extraversion, Openness-to-Experience, Agreeableness, and Conscientiousness), and multiple other personality variables (Andrei et al., 2016; Stamatopoulou et al., 2016; Chirumbolo et al., 2019). From these results, it is clear that the construct captures emotion-related variation neglected by frameworks such as the Big Five.

Because trait EI offers a comprehensive operationalization of the affective aspects of personality, it provides the most appropriate framework within which to investigate the general emotional functioning of people, including, of course, physicians. Moreover, trait EI correlates with many of the competencies that modern medical curricula seek to deliver (Arora et al., 2010; Andrei et al., 2016; Abe et al., 2018). Clinical ability in healthcare practice is crucial for treatment success and career prosperity, but incomplete without a trait EI basis (Lin et al., 2017; Sharp et al., 2020). To investigate trait EI in a medical context means to advance a holistic model that considers the inherent psychological features of physicians as applied to their clinical practice. It has been empirically shown that the trait EI profiles of physicians and nursing personnel may predict or mediate key outcomes in their professional life, such as job satisfaction (Hollis et al., 2017), burnout (Szczygiel and Mikolajczak, 2018), stress when undertaking surgical tasks (Arora et al., 2011; O’Connor et al., 2017), malpractice claims (Shouhed et al., 2019), and wellbeing (Lin et al., 2016). The burgeoning number of EI applications in the medical literature attests strongly to its importance in the field.

Gaps, however, remain. Most studies investigating trait EI within a medical setting have focused on medical competences or other professional outcomes for healthcare participants (Gupta et al., 2017; Abe et al., 2018; Al Huseini et al., 2019). What remains lacking is a systematic comparative analysis of trait EI profiles of medical providers against those of non-medical professionals. Such an analysis would be able to ascertain whether the trait EI profiles within healthcare are similar or dissimilar to those of professionals in other fields–hence pointing to the generalizability of trait EI applications (e.g., training) or, in contrast, to the need to tailor those specifically to the medical context.

Accordingly, the present study contributes toward addressing this gap in the evidence-base of trait EI within medicine. We evaluated the trait EI profiles of the following surgical specialties: General, Orthopedic, Head and Neck, and Miscellaneous surgical specialties. Moreover, we compared the surgical data against samples of distinct professions represented by engineers, executives and senior managers, lawyers, military managers (junior), nurses, and salespeople. We hypothesized that trait EI profiles would differ both within surgical specialties as well as between surgical specialties and other professions.

## Study 1

Study 1 compared the trait EI profiles of different surgical specialties using a leading multidimensional measurement instrument (Bru-Luna et al., 2021). Previous research that has looked at personality differences in healthcare samples has mostly taken the form of prospectively conducted surveys at medical schools (or equivalent), aimed at determining how personality might affect future specialty selection (e.g., Mullola et al., 2018). Moreover, cross-sectional studies across medical specialties have compared the personality profiles of different subgroups of specialized professionals against each other (e.g., Stienen et al., 2018). Taken together, the findings of these studies have proved hard to synthesize, and patterns of personality traits firmly associated with “personality types” across different specialties elusive to definitive descriptions.

We argue that one reason for this ongoing limitation in the field is the plethora of personality assessment tools used across studies based on theoretical approaches and psychometrics of variable quality. We further argue that the field suffers from a rather simplistic conception of a “personality type” across medical specialties, which assumes larger homogeneity within specialties than across them. Translated into a specific example, this assumption means that pediatric surgeons are expected to be “more like” orthopedic surgeons than pediatricians. This assumption is rarely tested empirically.

Study 1 aimed to address these shortcomings in the literature by directly measuring the trait EI of surgeons across several different surgical specialties. Due to the variability in the evidence on personality profiles of surgeons across different specialties; the variability in the theories and assessment tools applied; and the desirability of a combined surgical sample for subsequent analyses, we considered this study a necessary precursor to main Study 2.

## Materials and Methods

### Participants

One hundred and twenty-two surgeons participated in the study. The sample comprised 43 consultants (attending-level surgeons in the United States), 63 registrars (senior residents), and 18 core trainees (12 1st CT-year and 6 CT 2nd year; junior residents). Gender and age information are reported in Table 2. The following surgical specialties were represented in the sample: cardiothoracic (*n* = 5), general (*n* = 25), neurosurgery (*n* = 1), ophthalmology (*n* = 5), oral and maxillofacial (*n* = 4), orthopedic (*n* = 36), otolaryngology (*n* = 21), plastic (*n* = 6), urology (*n* = 11), and vascular (*n* = 4). Four surgeons listed their specialty as “other.”

**TABLE 2:** Gender and age information for the seven comparison samples in study 2.

*Occupation*	*Gender (M–F)*	*Age*
Engineers	27–3	40.28 (11.89)
Executives/senior managers	58–30	45.89 (7.18)
Lawyers	25–2	31.35 (6.59)
Military managers (junior)	61–6	29.43 (3.48)
Nurses	9–56	44.85 (8.58)
Salespeople	43–15	37.11 (9.15)
Surgeons	91–30	38.57 (8.90)

*One surgeon and five military managers did not report their gender. Numbers in parentheses are standard deviations.*

Following consultation with experienced academic surgeons, four surgical groups were created: General (*n* = 25), Orthopedic (*n* = 36), Head and Neck (comprising otolaryncology, ophthalmology, oral and maxilofacial, and neurosurgery; *n* = 31), and Miscellaneous (comprising cardiothoracic, vascular, plastics, urology, and other; *n* = 30) specialties.

### Measure

#### Trait Emotional Intelligence Questionnaire

The TEIQue is a 153-item inventory providing comprehensive coverage of the sampling domain of trait EI (Petrides, 2009). The instrument has shown excellent psychometric properties in multiple studies (Aluja et al., 2016; Andrei et al., 2016; Chirumbolo et al., 2019; Sanchez-Ruiz et al., 2021). Items are scored on a 7-point Likert scale and completion time is approximately 30 min. The 20 TEIQue variables (15 facets, 4 factors, and global trait EI) are presented in Table 1, along with brief explanations. All TEIQue instruments are available, free of charge, for academic and medical research purposes from www.psychometriclab.com.

### Procedure

Higher specialist trainees and consultants from all surgical specialties working in the former Northern Deanery region (now Health Education North East) of the United Kingdom were invited by email to take part in the study. Attached to the invitation was an information sheet detailing the aims of the study and the potential benefits and risks. This formed part of the consent process. Anonymity and the right to withdraw at any point without providing a reason were assured. The study received full ethical approval from the Camden and Islington Community Research Ethics Committee and was performed with the facilitation of the Northern Deanery. As a reward for their time, participants received a detailed personalized TEIQue feedback report (valued at $60) immediately on completion of the questionnaire, and were also entered into a prize draw for a netbook or shopping voucher worth $375. To improve recruitment, reminder emails were sent out on three occasions.

Participants were categorized into groups following receipt of their completed TEIQue forms. The grouping process aimed to produce clusters of participants that were conceptually as well as statistically viable. Grouping was led by an academic consultant surgeon (HCR) and done jointly with another study author (NS) based on consensus principles and with a view to achieving balanced group sizes across the surgical specialties.

Participants were clustered into the four aforementioned groups based on well-defined specialties (general and orthopedic), consideration of the part of the body they operate on (head and neck), and a final miscellaneous category to include specialties that could not be grouped otherwise due to small sample size (comprising cardiothoracic, vascular, plastics, urology, and other; *n* = 30).

## Results

A between-subjects analysis of variance with the four surgical groups as levels of the independent variable and global trait EI scores as the dependent variable was conducted. This returned a non-significant result [*F*_(3_, _118)_ = 0.66, *p* = ns]. A MANOVA at the factor level of trait EI (viz., with Wellbeing, Self-control, Emotionality, and Sociability as the DVs) was similarly non-significant [*F*_(12_, _304_._55)_ = 1.20, *p* = ns]. Corresponding analyses comparing trainee vs. consultant surgeons also returned non-significant results [ANOVA *F*_(1_, _120)_ = 2.68, *p* = ns: and MANOVA *F*_(4_, _117)_ = 1.55, *p* = ns].

## Discussion

This study revealed strong homogeneity in the trait EI profiles of surgeons. There were no differences between the groups, which spanned a range of surgical specialties, or between trainee (junior) and consultant (senior) surgeons. The latter finding is especially notable because of a potentially sizeable age gap between trainees and consultants, which might have expected to produce at least some differences in their profiles due to a small-to-moderate correlation between trait EI and age (Siegling et al., 2017; Aslanidou et al., 2018). No such differences were observed at either the global or the factor level of the construct.

Since the data are not longitudinal, we cannot be certain whether this homogeneity is the result of similarities at the entry point of surgical training or of a homogenization process operating during the course of training. Are the personalities of surgeons similar at the entry point of surgical training or do they gradually become similar as a result of training? In the absence of longitudinal data, it is worth highlighting that surgeons not only have their own reasons for entering surgical training, which differ from the reasons other medical doctors have for entering their own specialties, but that they are keenly aware of these reasons (Peel et al., 2018). Given that career choice is underpinned by clear-cut differences in personality (Farnia et al., 2018; Xin et al., 2020), it seems more likely that trainee surgeons have fairly homogeneous personalities upon entering medical training. This is further supported by the fact that there were no significant differences between trainee surgeons and consultants despite a potentially sizeable age difference between these two groups.

Although there is wide variation in perceptions of desirability and suitability of different medical careers, the personality characteristics of individuals *within* each medical specialty may be highly homogenous (McKinley et al., 2015). This seems to be the case as regards the affective aspects of the surgical specialty. Obviously, these findings cannot be automatically generalized to medical specialties other than those included in our research. Nevertheless, they are sufficiently robust to provide a ground for combining the present surgical data into a large group with a view to comparing it against pertinent professional groups.

## Study 2

Study 2 compared the trait EI profiles of surgeons against the profiles of professionals from a range of related (e.g., nurses) or socially comparable occupations (viz., occupations with similarly high social status, like lawyers and military managers). To our knowledge, this is the first such comparison in the field of emotional intelligence, and one of the more wide-ranging in the broader field of personality. Some relevant trait EI work was previously conducted by Siegling et al. (2014), who compared a sample of United Kingdom managers against the general population, Pérez-Díaz et al. (2021), who compared various public and private sector occupations, and Dugger et al. (2022), who compared a sample of U.S. pilots against the general population. None of these works involved any surgical specialties.

Other broadly relevant work presented a comparative analysis of the personality of surgeons based on a large sample obtained from an online personality assessment carried out by the BBC (Whitaker, 2018). Significant differences were reported between surgeons and non-surgeons on a Big Five tool, with the former scoring higher on conscientiousness, agreeableness, openness, and neuroticism. A limitation of that study was that the comparison sample was largely undifferentiated, when a comparison of surgeons against other professional groups could well carry specific implications for training and development.

Training approaches and modalities developed for high-risk industries, such as aviation or the military, have often been imported by surgical curricula without much adaptation or assessment of training needs and requirements, largely on the assumption that risk management and related skills are automatically transferrable across areas of professional activity (Kapur et al., 2016). Recently, such wholesale imports into surgery have been questioned and a more nuanced approach to surgical training has been called for (Gogalniceanu et al., 2021). Broader comparisons with socially comparable occupational groups, such as those included in this study, could provide support for such a nuanced approach.

Furthermore, differentiation between surgeons and other health professions, such as nurses, in terms of emotional predispositions and responsiveness can help tailor staff support and wellbeing programs that have rapidly proliferated since the advent of the COVID19 pandemic (e.g., Krausova et al., 2021). While such programs are a welcome addition to organizational staff support structures (and in many cases were needed even pre-pandemic; Laurino Neto and Herbella, 2019), tailoring them to professional groups’ needs can help increase perceived relevance and hence their uptake and effectiveness in supporting staff exposed to the stressors and pressures of the pandemic, and beyond.

Trait emotional intelligence is a strong predictor of job satisfaction, with effects that often persist over and above the Big Five and cognitive ability (Miao et al., 2017). It has also been found that leader trait EI relates positively to subordinate job satisfaction (Miao et al., 2016). Similarly, the construct has shown strong predictive ability in relation to job performance, again, over and above the Big Five and cognitive ability (e.g., O’Boyle et al., 2011). An ancillary aim of our study was to provide a first approximation of these effects in the domain of surgery.

### Materials and Methods

#### Participants

The surgeon sample described in Study 1 formed the focal (target) group in this study. As such, it was also the largest (*n* = 122). It was compared against 72 junior military managers, 30 engineers, 88 executives and senior managers in various roles (e.g., corporate management, human resources, and management consultants), 58 salespeople from various industries (e.g., retail, recruitment, and IT), 27 lawyers, and 65 nurses from a broad range of specialties and roles (e.g., junior and senior sisters, midwives, and directors of nursing departments). All groups were sufficiently large to enable parametric comparisons as per the central limit theorem. Gender and age details about these groups are given in Table 2.

#### Measures

##### Trait Emotional Intelligence Questionnaire

As described in Study 1.

##### Job Satisfaction and Job Performance

Responses to single-item measures of job satisfaction and perceived job performance were available for all groups, except lawyers. These questions were answered on 7-point Likert scales and their exact phrasing was “How happy in your job are you?” and “How good are you at your line of work?”

#### Procedure

The collection process of the surgical data was described in Study 1. Military data were collected from the Army Training Regiment in Winchester (England) from Corporals, Sergeants, and Officers. Additional data were retrieved from the TEIQue archives. All questionnaires were completed individually and voluntarily. Completion took approximately 30 min.

## Results

### Occupational Differences in Trait Emotional Intelligence

A between-subjects analysis of variance with the seven occupational groups as levels of the independent variable and global trait EI scores as the dependent variable was performed. There was a significant main effect of occupational group [*F*_(6,50)_ = 19.38, *p* < 0.01, η^2^ = 0.21]. This was followed through with a series of Dunnett’s *post-hoc* tests with surgeons set as the comparison (target) group. These comparisons, whose details are presented in Table 3, showed that surgeons scored significantly higher on global trait EI than junior military managers, but lower than executives and senior managers, salespeople, and nurses. There were no significant differences in the comparisons against engineers and lawyers.

**TABLE 3:** Comparisons (Dunnett *t*-tests) between surgeons and six professional groups.

	*N*	*Mean Difference*	*Std. Error*
Engineers	30	0.031	0.112
Executives/Senior managers	88	0.502*	0.076
Lawyers	27	0.016	0.116
Military managers (junior)	72	−0.278*	0.083
Nurses	65	0.289*	0.084
Salespeople	58	0.499*	0.087

Dunnett t-tests treat one group as a control (Surgeons; n = 122) against which all other groups are compared. Comparisons are based on a 7-point Likert scale. *p < 0.01.

To investigate these data at the factor level of trait EI, a MANOVA was conducted with the seven occupational groups as levels of the independent variable, and the four trait EI factors (Wellbeing, Self-control, Emotionality, and Sociability) as the dependent variables. There was a significant multivariate main effect of occupational group, *F*_(24_, _1560_._61)_ = 6.66, *p* < 0.001, η_*p*_^2^ = 0.08. Follow-up ANOVAs revealed highly significant differences on all four trait EI factors: Wellbeing, *F*_(6,450)_ = 10.13, *p* < 0.001, η_*p*_^2^ = 0.12; Self-control, *F*_(6,450)_ = 10.13, *p* < 0.001, η_*p*_^2^ = 0.16; Sociability, *F*_(6,450)_ = 10.76, *p* < 0.001, η_*p*_^2^ = 0.13; and Emotionality, *F*_(6,450)_ = 15.70, *p* < 0.001, η_*p*_^2^ = 0.13.

Dunnett’s *post-hoc* tests indicated that surgeons scored significantly higher (*p* < 0.01) than junior military managers on Wellbeing and Self-control. Conversely, they scored significantly lower than executives and senior managers on all four trait EI factors (*p* < 0.01), lower than salespeople on Self-control, Emotionality, Sociability (*p* < 0.01), and marginally lower (*p* = 0.05) on Wellbeing. Surgeons also scored lower than nurses on Emotionality (*p* < 0.01).

### Correlations With Criteria

As an indicator of the impact of trait EI on job satisfaction and job performance, we estimated within-group correlations. Very interestingly, the correlations of global trait EI with job satisfaction and job performance were highest for surgeons (*r*_*jobsat*_ = 0.467 and *r*_*jobper*_ = 0.457) and second-highest for nurses (*r*_*jobsat*_ = 0.417 and *r*_*jobper*_ = 0.448).

All four trait EI factors (Wellbeing, Self-control, Emotionality, and Sociability) predicted both job satisfaction and job performance. The strongest correlations at the factor level were for Wellbeing and Sociability. The full correlation matrix is depicted in Table 4.

**TABLE 4:** Correlations between Trait EI (Global and Factor Scores), job satisfaction, and job performance in surgeons.

	1	2	3	4	5	6	7
1. Wellbeing	–	0.46*	0.48*	0.61*	0.81*	0.56*	0.42*
2. Self-control		–	0.38*	0.41*	0.70*	0.29*	0.35*
3. Emotionality			–	0.56*	0.81*	0.27*	0.27*
4. Sociability				–	0.79*	0.31*	0.44*
5. Global trait EI					–	0.47*	0.46*
6. Job satisfaction						–	0.39*
7. Job performance							–

*p < 0.01.

## Discussion

The analyses revealed sharp trait EI profile differences between surgeons and several other professional groups. There were no differences vis-a-vis lawyers and engineers, however, surgeons scored significantly higher on global trait EI than junior military managers, and lower than executives and senior managers, salespeople, and nurses. These differences were broadly reflected across the four trait EI factors too.

With respect to junior military managers, it should be noted that some of the relevant variance may be confounded by age. The literature suggests that trait EI increases, at least moderately, with age (Tsaousis and Kazi, 2013; Pérez-Díaz et al., 2021), and so the nine-year gap between surgeons and junior military managers (the largest in the dataset; see Table 2) could partially explain the difference in trait EI means. Nevertheless, these differences generally persisted, albeit slightly diminished, in ANCOVAs covarying out age. Therefore with some caution, because the age correction does not also correct for the fact that military managers were drawn from the junior levels of the army, we can conclude that surgeons have significantly higher trait EI scores. This finding probably reflects the necessity in military settings of keeping the emotional aspects of personality in check. It echoes findings based on airline pilots, whose training and organizational cultures share many similarities with those of military managers (Dugger et al., 2022).

Executives and senior managers, salespeople, and nurses, all scored higher than surgeons on global trait EI, and in most comparisons, on the four trait EI factors too. From the perspective of surgeons, the key question is whether relatively lower levels of trait EI are conducive to their work life. There is a salient interpersonal aspect affecting both the teamworking skills that surgeons need to show while in the operating theater (Tørring et al., 2019) as well as their interactions with patients and their families (Hsu et al., 2019). “Soft” skills in surgeons, which are mirrored across multiple trait EI facets, may contribute to enhanced patient care and improved relationships with colleagues and staff (Sharp et al., 2020). In addition, other personality traits that are important in surgery, and more broadly in medicine, such as emotion regulation and acute stress management, are specifically linked to intrapersonal facets of trait EI (Weilenmann et al., 2018; Grantcharov et al., 2019). Overall, surgeons experience high levels of stress, burnout, and suicidal ideation (Shanafelt, 2011; Dimou et al., 2016), which is why trait EI training can play a central role in improving their ability to cope with pressure and prevent or ameliorate burnout (Lindeman et al., 2017; Sharp et al., 2020).

Enhancements in the psychological wellbeing of surgeons will likely be reflected in their patients’ satisfaction levels. Effective healthcare is often measured in terms of patient satisfaction, which is considered not just the element of care delivered to patients, but also the way in which this was received and experienced by the patients during the episode (Sharp et al., 2020). It seems important to identify how, and under what circumstances, trait EI may impact on major aspects of care delivery and, subsequently, use this information to amend existing interventions or develop new ones explicitly tailored to surgeons. Trait EI-tailored interventions have shown substantial and consequential score gains in experimental research designs with university students (see Nelis et al., 2009, 2011) as well as in naturalistic research in schools (Ruttledge and Petrides, 2012; Li and Xu, 2019). Related research has shown that other types of intervention, like Yoga training, can also lead to score increases in trait EI measures (McIlvain et al., 2015).

The applicability of trait EI in the work-life of surgeons is on full display in the strong positive correlations between trait EI scores and ratings of job satisfaction and job performance. Undoubtedly, these results should be replicated with fully validated measures of job satisfaction and job performance, although it is worth noting that single-item measures may, under certain circumstances, offer a viable psychometric alternative that balances demands of brevity with those of reliability and validity. Technically, single-item measures are prone to underestimate the relationships between constructs (Credé et al., 2012), which means that trait EI may be even more strongly related to job satisfaction and job performance in surgeons than suggested by our findings. However, we recognize our reliance on single-item measures as a limitation in Study 2 and reiterate our call for replication with fully validated criterion measures.

In addition to the aforementioned issue with the single-item criterion measures, the study has further limitations. There is a self-selection bias inherent to most studies that rely on survey methodologies. Since we have no means of testing the populations of interest, we cannot rule out the possibility that our participants self-selected based on their interest in the topic. Another limitation concerns the relatively small sample sizes of the design. While we are confident that the study was sufficiently powered to detect large and medium-sized effects, future research could endeavor to replicate these results with larger samples, additional surgical specialties as well as additional occupational groups. We explicitly recognize the existence of EI paradigms other than trait EI (e.g., ability EI by Mayer and Salovey, 1997 or the behavioral model by Boyatzis, 2009) and caution that an altogether different pattern of results and/or interpretations may be observed within those paradigms. These limitations do not detract from the strengths of the study, which presents rare trait EI data across multiple surgical specialties but also with reference to other professional groups, and statistical comparisons down to the factor level of the construct using a leading multidimensional measurement instrument.

Detailed assessment of surgeons’ emotional perceptions is feasible and allows for comparative analyses within surgery, but also between surgery and other occupations. As per the expanding literature on trait EI, these perceptions correlate with several aspects of surgical careers, including job-related outcomes, stress management strategies, and resilience levels. This line of research can help with the tailoring of surgical training curricula across several stages, from post-graduate education to continuing professional development. It can also facilitate the design, implementation and uptake of staff support and wellbeing programs through effective customization for surgical populations. The current COVID19 pandemic means such tailoring is an urgent need, though the literature suggests it is also a long-standing necessity to support surgeons’ successful and holistic career management. Future studies should explore surgeons’ trait emotional intelligence in larger samples, and possibly longitudinally so as to trace potential changes due to aging and generally increasing professional experience, while also carrying out similar assessments in non-surgical physicians.

## Data Availability Statement

The raw data supporting the conclusions of this article will be made available by the authors, without undue reservation.

## Ethics Statement

The study received full ethical approval from the Camden and Islington Community Research Ethics Committee and was performed with the facilitation of the Northern Deanery. The patients/participants provided their written informed consent to participate in this study.

## Author Contributions

KP, MP, PP-D, SJ, HR, NS, and NA performed the material preparation and collected and analyzed the data. KP and NS wrote the first draft of the manuscript. All authors contributed to the study conception and design, commented on previous versions of the manuscript, and read and approved the final manuscript.

## Author Disclaimer

The views expressed in this publication are those of the authors and not necessarily those of the NIHR, the ESRC, the charity, Department of Health and Social Care, CNPq or the HJEMFA.

## Conflict of Interest

SJ was employed by company Steve Jeffrey International FZE LLC. NS was the director of the London Safety and Training Solutions Ltd., which offers training in patient safety, implementation solutions and human factors to healthcare organizations. The remaining authors declare that the research was conducted in the absence of any commercial or financial relationships that could be construed as a potential conflict of interest.

## Publisher’s Note

All claims expressed in this article are solely those of the authors and do not necessarily represent those of their affiliated organizations, or those of the publisher, the editors and the reviewers. Any product that may be evaluated in this article, or claim that may be made by its manufacturer, is not guaranteed or endorsed by the publisher.
